# Taxonomically Restricted Genes with Essential Functions Frequently Play Roles in Chromosome Segregation in *Caenorhabditis elegans* and *Saccharomyces cerevisiae*

**DOI:** 10.1534/g3.117.300193

**Published:** 2017-08-24

**Authors:** Adrian J. Verster, Erin B. Styles, Abigail Mateo, W. Brent Derry, Brenda J. Andrews, Andrew G. Fraser

**Affiliations:** * The Donnelly Centre, University of Toronto, Ontario M5S 1A8, Canada; † Department of Molecular Genetics, University of Toronto, Ontario M5S 1A8, Canada; ‡ Developmental and Stem Cell Biology Program, The Hospital for Sick Children, Peter Gilgan Centre for Research and Learning, Toronto, Ontario M5G 0A4, Canada

**Keywords:** chromosome segregation, *Saccharomyces cerevisiae*, *Caenorhabditis elegans*

## Abstract

Genes encoding essential components of core cellular processes are typically highly conserved across eukaryotes. However, a small proportion of essential genes are highly taxonomically restricted; there appear to be no similar genes outside the genomes of highly related species. What are the functions of these poorly characterized taxonomically restricted genes (TRGs)? Systematic screens in *Saccharomyces cerevisiae* and *Caenorhabditis elegans* previously identified yeast or nematode TRGs that are essential for viability and we find that these genes share many molecular features, despite having no significant sequence similarity. Specifically, we find that those TRGs with essential phenotypes have an expression profile more similar to highly conserved genes, they have more protein–protein interactions and more protein disorder. Surprisingly, many TRGs play central roles in chromosome segregation; a core eukaryotic process. We thus find that genes that appear to be highly evolutionarily restricted do not necessarily play roles in species-specific biological functions but frequently play essential roles in core eukaryotic processes.

The genomes of several hundred diverse eukaryotes have been sequenced. Comparing these genomes reveals sets of highly conserved, ancient genes that have clear orthologs present across the phylogenetic tree; these genes are typically well characterized and encode components of core eukaryotic machineries. For example, almost all eukaryotes have highly conserved orthologs of genes encoding components of the ribosome, proteasome, and nuclear pore. We use the term “shared gene” to refer to these genes which are shared across the phylogenetic tree. At the other end of the spectrum are a much more enigmatic and poorly studied set of genes, the large numbers of genes that have no detectable homology outside a very small set of highly related species. Such genes are found in the genome of every new species examined (reviewed in Tautz and Domazet-Lošo 2011 and Chen *et al.* 2013) and frequently make up a large proportion of encoded genes; for example, ∼10–20% of protein-coding genes in any typical eukaryotic genome have no detectable homology outside closely related species (Khalturin *et al.* 2009). Furthermore, as more genomes are sequenced, the number of these poorly conserved genes is ever increasing (Wilson *et al.* 2005) and they can be identified even when closely related species are compared. For example, human-specific genes have been identified that are not present in chimpanzees (Knowles and McLysaght 2009). These genes were initially termed “orphan genes” (Dujon 1996) and are more formally referred to as taxonomically restricted genes (TRGs); we use this terminology throughout.

Many mechanisms have been proposed to explain the origin of TRGs, and these fall into two broad categories. The first way is new gene birth. Here, new TRG open reading frames can arise directly from previously noncoding DNA: these *de novo* TRGs are entirely novel at their moment of birth. This process of new gene birth occurs frequently and gives rise to many new functional genes (Carvunis *et al.* 2012; Zhao *et al.* 2014). The alternative route for TRG creation is that they can arise from existing genes by mechanisms such as gene duplication, retrotransposition or recombination resulting in chimeric genes, or by the extreme divergence of an ancestral gene. In these cases, the TRGs may carry some or all of the functions of the ancestral genes from which they derive. Whether TRGs arise from previous noncoding DNA or through massive changes to ancestral coding sequences, TRGs encode proteins that sample novel areas of protein sequence space and thus could encode novel functions. However, despite extensive work examining the birth and origin of TRGs (Tautz and Domazet-Lošo 2011; Chen *et al.* 2013), we know very little about their functions. TRGs typically contain no characterized domains, have no detectable homology outside the genomes of highly related species, and are almost entirely neglected in traditional hypothesis-driven experiments. What do they do? In particular, do TRGs have functions specific to the particular biology of the species, or do TRGs play roles in conserved molecular processes? For example, do human-specific TRGs have human-specific functions or do human TRGs carry out conserved molecular functions in a human-specific way? While there are many examples of the biological roles for individual TRGs, ranging from novel biosynthetic pathways (Weng *et al.* 2012), adaptation to cold environments (Chen *et al.* 1997b), and honeybee eusociality (Johnson and Tsutsui 2011), there have been no systematic studies to directly examine TRG functions in tractable model organisms. Our goal in this study is to use the systematic gene function data sets available in the budding yeast *Saccharomyces cerevisiae* and the nematode worm *Caenorhabditis elegans* to investigate the functions of TRGs and how these evolved.

## Materials and Methods

### Defining TRGs

To define *S. cerevisiae* and *C. elegans* TRGs, we employed a method similar to the “phylostratum” approach (Domazet-Lošo and Tautz 2010). In brief, we defined *C. elegans* TRGs as genes with no evidence of homology outside Chromadorea; this includes *Pristionchus pacificus*, which diverged ∼300–400 MYA (Dieterich *et al.* 2008). For *S. cerevisiae*, we identified TRGs as genes with no homology outside Saccharomycetales, close to the most recent common ancestor of *S. cerevisiae* with *Candida albicans*, which leaves us with a comparable divergence time of 100–300 MY of evolution (Taylor and Berbee 2006). To assess homology, we took the proteome of either species [*C. elegans* (WS230) and *S. cerevisiae* version 2011203] and used BLASTP to find genes with sequence similarity in the NCBI nonredundant protein database (February 7, 2013). Any BLASTP hit with an *E*-value <0.000001 over >30% of the protein length was considered evidence of homology, though we note that we tested other cutoffs and found that the analyses of this article were qualitatively similar (data not shown). In addition, we excluded any putative TRGs that encoded a known domain, as identified in the InterPro database (Mitchell *et al.* 2014). *C. elegans* TRGs thus only have homology to genes in Chromadorea, *S. cerevisiae* TRGs only have homology to genes in Saccharomycetales, and no TRGs contain any conserved domains.

The phylostratum approach splits genes into groups depending on where on the tree of life they appear to have emerged, while instead we take a much coarser approach of a single group of TRG proteins. Since our article focuses on comparing essential and nonessential TRGs, and since there are very few essential TRGs, we cannot use a finer gradation on TRG age as it would eliminate our statistical power.

We defined an essential gene in *C. elegans* as those genes with either nonviable or growth-defective phenotypes in genome-wide RNA-mediated interference (RNAi) screens (Kamath *et al.* 2003), since these are the most robust phenotypes. In *S. cerevisiae* we used the set of essential genes identified by the *Saccharomyces* Genome Deletion Project (Giaever *et al.* 2002).

### The expression of genes in the region surrounding TRGs

We examined the expression of genes using RNA-Seq data from young adult-staged *C. elegans* worms (Ramani *et al.* 2009) in the genes which flanked genes of interest. To examine chromatin state, we obtained ChIP-chip data from modEncode (Liu *et al.* 2011) and calculated the median signal up to 5 kb upstream and 5 kb downstream of each gene of interest. In our analysis, we examined H3K27 methylation as a representative repression mark and H3K79 methylation as a representative activation mark. In all cases we tested for significance between different groups of genes using a Wilcoxon rank-sum test.

### The expression correlation between TRGs and different molecular pathways

We measured expression correlation using a curated and normalized set of microarray data sets from the WormSPELL database. We calculated the Pearson correlation coefficient for a given microarray between the expression of a TRG and the expression of every other gene represented on the array. To improve the comparability of correlation coefficients between data sets, we used Fisher’s Z-transformation (Hibbs *et al.* 2007), and then averaged the correlations across arrays. For each TRG we further combined the correlation values to genes within a given KEGG pathway and averaged them to create a single number representing the correlation of this TRG to that group of genes. This yielded a single value which represents the average correlation of a given TRG to genes in a pathway of interest, averaged across a large data set of microarrays.

We used these correlation values in a logistic regression classifier, regularized with an elastic net (α = 0.5). We measured the strength of our classifier using area under the receiver operating characteristic (ROC) curve in cross-validation. To assess which features were most important for classification accuracy, we tested each feature individually in the logistic regression model using this same area under the ROC curve metric.

### The number of protein–protein interactions made by TRGs

Worm protein–protein interaction (PPI) data were downloaded from Worm-Interactome 8 (Simonis *et al.* 2009), yeast PPI data were from the BioGRID database (Stark *et al.* 2006), and we only examined genes with at least one interaction. We clustered the yeast PPI network using the Markov Clustering Algorithm (MCL) (Enright *et al.* 2002), with an inflation factor of 1.5. We tested all clusters with >30 members for enrichment in the number of TRGs using a hypergeometric test, and corrected for multiple tests using the Bonferroni correction.

### The predicted level of protein disorder in TRGs

To estimate the amount of protein disorder in genes of interest, we predicted the fraction of each protein which is ordered using Espritz (Walsh *et al.* 2012), which uses a neural network trained on data from Protein Data Bank. For each protein, we calculate the fraction of residues which are ordered and established a statistical difference using a Kolmogorov–Smirnov test. Coiled-coil regions were predicted using paircoil2 (Mcdonnell *et al.* 2006).

### Yeast protein evolution

We took dN/dS values for *S. cerevisiae* genes from Scannell *et al.* (2011), and functional annotations from Costanzo *et al.* (2010). We examined the distribution of dN/dS for each functional class and tested for significant increased/decreased rates of divergence relative to all predicted *S. cerevisiae* genes with a *t*-test.

### Worm microscopy

Chromosome-segregation defects will be visible at the earliest cell divisions in development, therefore, we imaged early embryos of the OD95 *C. elegans* strain (Essex *et al.* 2009) with mCherry-tagged histone (HIS-58) and a GFP tagged with the PH domain of PLC1Δ1, which allowed us to visualize the plasma membrane. RNAi was administered by feeding to L1-staged worms and their embryos (F_1_ generation) were visualized for cell-division defects using a Leica DMRA compound microscope equipped with epifluorescence (100× lens). As a negative control, we used a nontargeting RNAi in the form of *Escherichia coli* strain HT115 expressing the RNAi plasmid vector L4440. As a positive control, we used an RNAi construct targeting *hcp-3* which is the homolog of CENP-A. The genotype of strain OD95 is: *unc-119*(*ed3*) III; *ltIs37* [pAA64; P*pie-1*::mCHERRY::*his-58*; *unc-119*(*+*)] IV; *ltIs38* [pAA1; P*pie-1*::GFP::PH(PLC1Δ1); *unc-119*(*+*)]).

### Yeast microscopy

We obtained temperature-sensitive yeast strains from Li *et al.* (2011) and Ben-Aroya *et al.* (2010). These were crossed to a *MAT*α strain harboring endogenously tagged *DAD2-GFP*, which enables us to visualize chromosome segregation, and *HTA2-mCherry* as well as the *RPL39* promoter driving the constitutive expression of tdTomato, which allow us to visualize the cell morphology by marking the nucleus and cytosol. *MAT***a** haploid segregants were obtained by random sporulation (as described in Sherman *et al.* 1979). These haploid strains were then grown to saturation in SD medium without histidine, uracil, leucine, arginine, and lysine, and in the presence of nourseothricin (# CAS 96736-11-7; Werner BioAgents) and Geneticin (# 11811098; Life Technologies), and then subcultured and grown to midlog phase in medium lacking the same suite of amino acids and in the presence of the same drugs, but containing low fluorescent yeast nitrogen base (#4030-512; MP Biomedicals LLC). Log-phase cells were incubated at a restrictive temperature of 37° for 3 hr before being imaged using a spinning disc confocal system (WaveFX; Quorum) on a Leica DMI6000B Microscope with Volocity 4 software (PerkinElmer).

### Data availability

Supplemental Material, Table S1, contains a list of TRGs from *C. elegans* and *S. cerevisiae* and includes whether they are essential. File S1 contains the supplemental figure legends.

## Results

**Figure 1:**
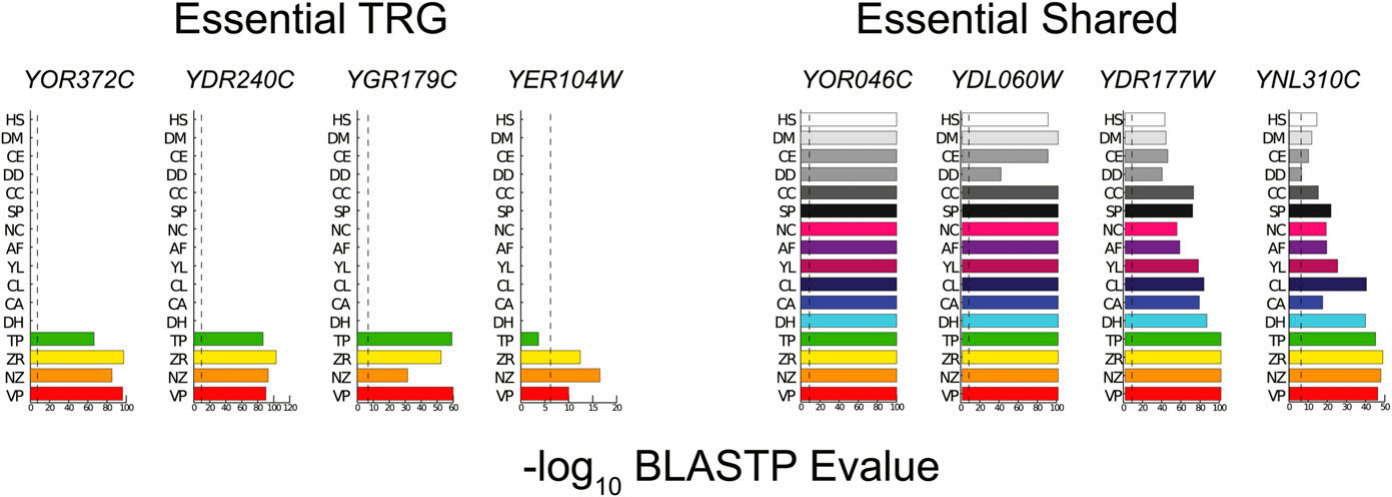
Phylogenetic distribution of BLAST scores for four essential TRGs and four shared essential genes. Each plot shows the −log_10_ BLASTP *E*-value for the highest BLAST match of each *S. cerevisiae* gene in a range of eukaryote genomes, including those of the closely related yeasts *Vanderwaltozyma polyspora* (VP), *Naumovia castelli* (NC), *Zygosaccharomyces rouxii* (ZR), *Tetrapisispora phaffii* (TP), *Debaryomyces hansenii* (DH), and *C. albicans* (CA); more distantly related yeasts *Clavispora lusitaniae* (CL), *Yarrowia lipolytica* (YL), *Aspergillus fumigatus* (AF), and *S. pombe* (SP); as well as the fungus *Coprinopsis cinerea* (CC); the slime mold *Dictyostelium discoideum* (DD); and the animals *C. elegans* (CE), *Drosophila melanogaster* (DM), and *Homo sapiens* (HS). Genomes are ordered bottom to top from closest to furthest from *S. cerevisiae*, and the dotted line marks the cutoff used to define TRGs. Similar plots for all yeast TRGs can be found in Figure S2.

We first identified all TRGs in either the *S. cerevisiae* or *C. elegans* genome by examining the conservation of all *S. cerevisiae* or *C. elegans* genes across the tree of life. This approach is widely used to identify TRGs (Tautz and Domazet-Lošo 2011; Chen *et al.* 2013). We identified *S. cerevisiae* genes that are taxonomically restricted to the Saccharomycetales lineage (we refer to these as “yeast TRGs”) or *C. elegans* genes restricted to the Chromadorea lineage (we term these the “worm TRGs”). Finally, we also excluded any putative TRGs that contain well-annotated protein domains (see *Materials and Methods*). This led us to define 691 yeast TRGs and 6018 worm TRGs (listed in Table S1). We note that even when we greatly relax our criteria for detecting homology, we fail to find any matches outside the restricted lineages for the great majority of the TRGs (see Figure S1). For example, >75% of yeast TRGs examined have no matches outside yeast genomes, even using the excessively relaxed criteria of 5% length match with a BLAST score of 10^−2^. Consistent with this, while BLAST scores of many shared genes fall off slowly with phylogenetic distance, they drop off precipitously for TRGs (examples in Figure 1 and for all yeast TRGs studied in Figure S2); there are no significant hits outside a narrow set of closely related species. In summary, the TRGs we identify have no significant homology outside the chosen lineages and have no protein domains that are found outside the chosen lineages. These TRGs thus encode novel proteins that in turn have the potential to carry out novel functions. What are they?

Crucially for our study, there is extensive functional data on all predicted *S. cerevisiae* and *C. elegans* genes. In particular, systematic studies of gene function have found ∼20% of yeast genes (Giaever *et al.* 2002) and ∼15% of worm genes (Kamath *et al.* 2003) to be absolutely required for organism viability; for reasons of brevity, we and others term these “essential genes.” While many of the other yeast and worm genes can also affect fitness (Hillenmeyer *et al.* 2008; Ramani *et al.* 2012), the essential genes have the most profound impact on fitness and are distinct in many ways. They are required for viability in almost all conditions, are under stronger negative selection than other genes, and they are more highly transcribed (*14*–*16*, Figure S3). Most essential genes in yeast and worm are orthologs (Kamath *et al.* 2003; Tischler *et al.* 2006) encoding components of basic cellular machineries such as the ribosome and the proteasome (Zotenko *et al.* 2008), and we refer to these shared essential genes as “shared” essentials throughout. Intriguingly, however, a small proportion of the essential genes, 23 (3%) in yeast and 96 (2%) in worm, are either yeast-specific or worm-specific TRGs. These TRGs have functions as central to the organism as shared cellular processes such as protein translation—what are these biological roles? In the rest of this article, we explore the differences between these essential TRGs and the other, nonessential TRGs that give us insight into how a TRG might be as essential for the viability of an organism as a shared gene. We examine in turn three aspects of gene function: where and when these genes are expressed, the physical interactions made by the novel encoded proteins, and their *in vivo* functions.

### Essential TRGs have similar expression to shared essential genes

**Figure 2:**
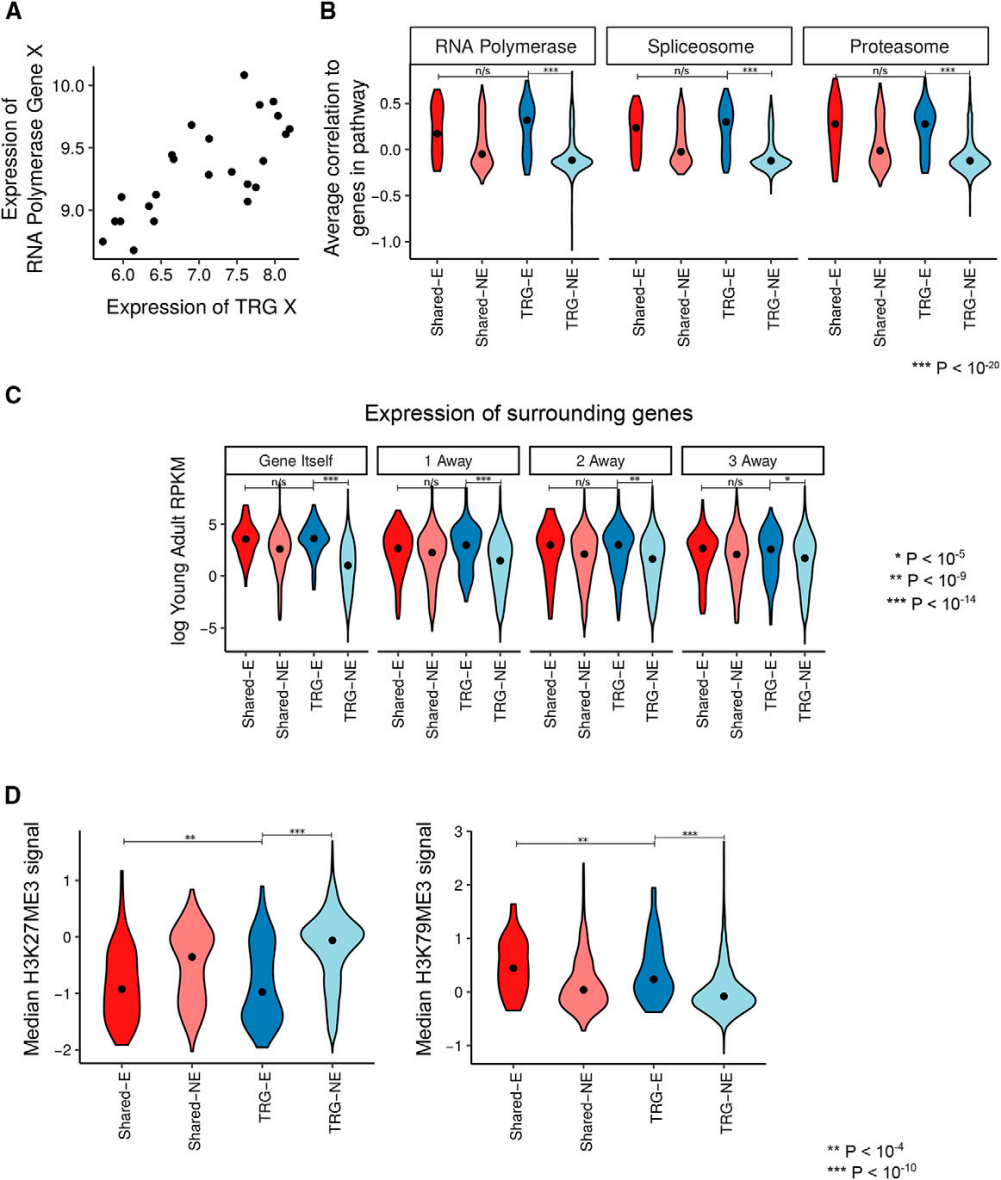
TRGs with essential functions are differently expressed compared to nonessential TRGs. (A) Example of the relationship between the expression of a TRG and a gene from the RNA polymerase. Here we show the expression in a single data set from WormSPELL. The essential TRG is WBGene00020105 and the gene from RNA polymerase is WBGene00017830. (B) Distribution of expression correlations between *C. elegans* TRGs and conserved KEGG pathways. Here we show the distribution of the expression correlations between groups of genes of interest and cel03020 (RNA polymerase II), cel03040 (the spliceosome), or cel03050 (the proteasome). In all cases, essential TRGs show higher correlations than nonessential TRGs. Correlations have been Z-transformed to improve comparability between data sets. (C) Essential TRGs tend to be surrounded by highly expressed genes. We examined the *C. elegans* young adult expression levels of different groups of genes. This is shown in the box plot “Gene Itself”; box denotes the interquartile range (IQR). In addition, we assessed expression levels of flanking genes (“1 away,” “2 away,” and “3 away”) of different groups of genes. *P*-values show the significance of the difference between essential TRGs and nonessential TRGs, or between essential TRGs and shared essentials. (D) Essential TRGs tend to be located in regions of active chromatin. We calculated the median level of either H3K27Me3 (repressive) or H3K79Me3 (active) marks in a 5-kb region centered on each gene in the genome. The box plots show the distribution of signals for each gene class; boxes denote IQR. As above, *P*-values show the significance of the difference between essential TRGs and nonessential TRGs, or between essential TRGs and shared essentials. Shared-E, essential shared genes; Shared-NE, nonessential shared genes; TRG-E, essential TRGs; TRG-NE, nonessential TRGs.

We then first examined whether there were any differences in gene expression between essential TRGs and nonessential TRGs using existing data sets. It is well known that genes whose expression is correlated tend to have similar functions (Hughes *et al.* 2000). We thus explored the similarity in expression profiles between TRGs and genes with well-established functions across a wide array of different expression data sets (Figure 2A). Specifically, we divide genes into specific functional modules using annotations from KEGG (*Materials and Methods*). For each TRG, we calculated its average coexpression with each KEGG-annotated functional module and find that essential TRGs tend to have more similar expression profiles than nonessential TRGs to shared modules such as RNA polymerase and the proteasome (Figure 2B for *C. elegans* and Figure S4 for *S. cerevisiae*), suggesting that essential genes have similar expression regardless of level of conservation. Finally, we examined whether combining all of these measurements of expression correlation together can predict whether a TRG is likely to be essential. We used logistic regression regularized with an elastic net (*Materials and Methods*) and find that expression correlation between a TRG and different KEGG pathways is strongly predictive of TRG essentiality (this yields an area of 0.87 under the ROC curve, Figure S5A). We note that not all KEGG modules make an equal contribution to these predictions, specifically those modules comprised of essential genes are playing a disproportionate role (Figure S5B). This suggests that essential TRGs are expressed in similar ways to shared essentials across a wide variety of different conditions and developmental stages.

In *C. elegans*, there are significant correlations in expression profile and level between neighboring genes: genes do not appear to be entirely independent transcriptional units, but large chromosomal domains appear to share similar expression characteristics (Roy *et al.* 2002; Kamath *et al.* 2003). We find that the gene neighbors of essential TRGs tend to have higher expression levels than the gene neighbors of nonessential TRGs (Figure 2C), and the chromatin in the genomic region surrounding essential TRGs tends to be more transcriptionally active than that surrounding nonessential TRGs (Figure 2D); these trends are most marked in *C. elegans*. This suggests that the transcriptional environment surrounding the genomic location of a TRG may play a key role in its expression and hence in its ability to carry out an essential function.

In summary, then, we find that essential TRGs have similar expression profiles to shared essential genes, whereas nonessential TRGs have different expression profiles. At least at the level of expression, essential genes look similar regardless of their level of conservation across species.

### Taxonomically restricted proteins tend to interact with shared essential proteins

We next examined the physical interactions made by proteins encoded by TRGs; we term these taxonomically restricted proteins (TRPs) for brevity. TRPs are known to have a different spectrum of PPIs when compared to shared genes (Capra *et al.* 2010); here we compare the PPI characteristics of essential TRPs compared to nonessential TRPs. We note that some analyses were only possible in yeast protein interaction networks where the coverage is far greater than for worm proteins; the yeast protein interaction network we use comprises >55,000 interactions and includes >75% of all yeast proteins, whereas the largest *C. elegans* protein interaction network has only ∼4300 interactions covering only ∼15% of worm proteins (*Materials and Methods*).

**Figure 3:**
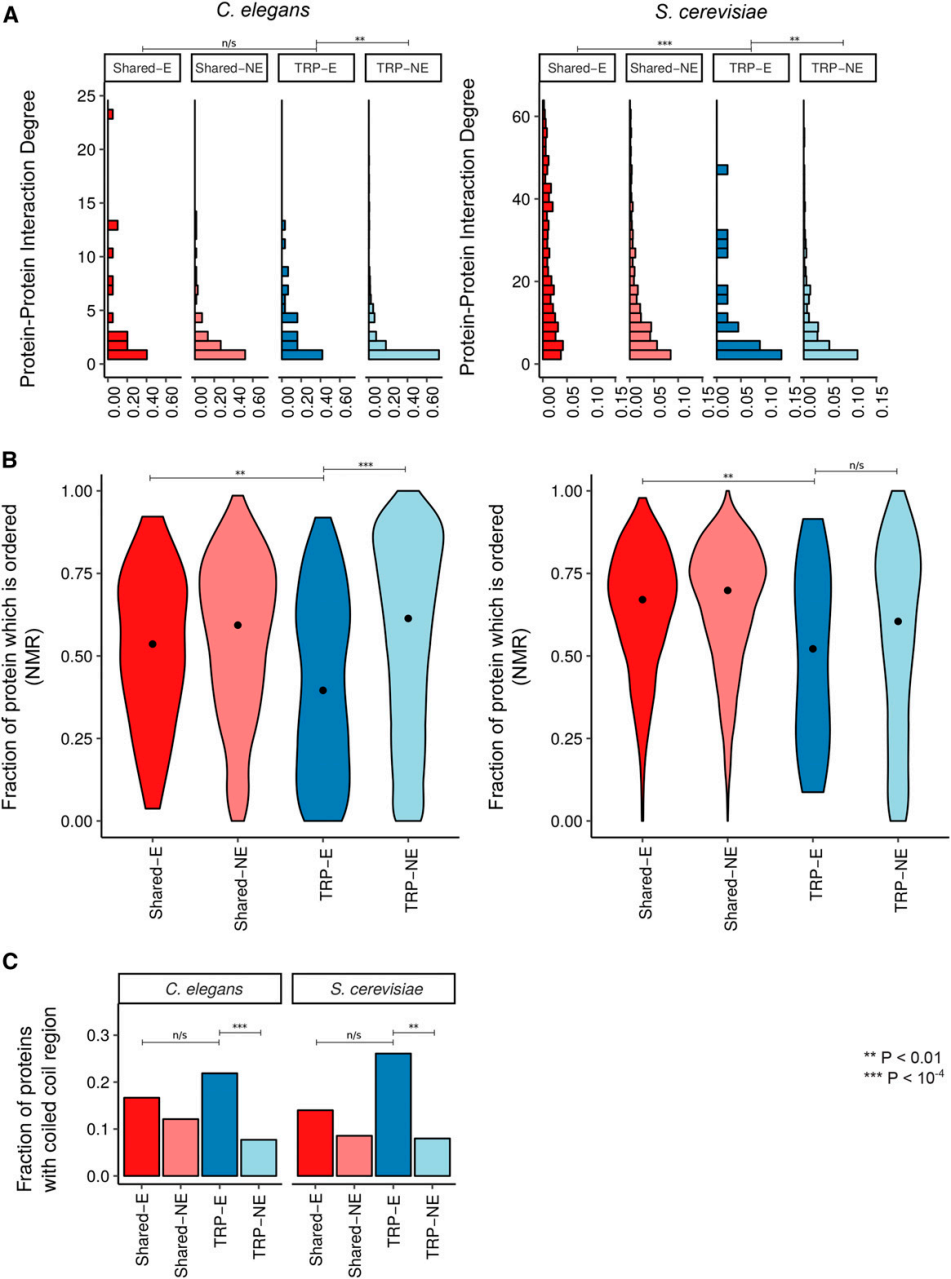
PPIs are significantly different between essential and nonessential TRPs. (A) Density plots of the number of protein–protein physical interactions. *S. cerevisiae* interactions are AP-MS data from BioGRID. *C. elegans* interactions are the interactions from Worm Interactome 8 (Simonis *et al.* 2009) with a minimum of one interaction. The * indicates significant *P*-values from a Wilcoxon rank-sum testing for the difference between essential and nonessential TRPs, or between essential TRPs and shared essential proteins. (B) Predicted protein disorder in TRPs and shared proteins in *C. elegans* and *S. cerevisiae*. We predicted the fraction of each protein which was ordered with protein sequence alone with Espritz (Walsh *et al.* 2012). We show violin plots of NMR mobility disorder and significance is assessed using a Kolmogorov–Smirnov test. (C) Fraction of proteins that contain a coiled-coil region. We plot the fraction of proteins that contain a coiled coil as predicted from protein sequence using paircoil2 (Mcdonnell *et al.* 2006). The * indicates significant *P*-values from a Wilcoxon rank-sum testing for the difference between essential and nonessential TRPs, or between essential TRPs and shared essential proteins. Shared-E, essential shared proteins; Shared-NE, nonessential shared proteins; TRP-E, essential TRPs; TRP-NE, nonessential TRPs.

Previous studies showed that proteins encoded by essential genes have more interactions than those encoded by other genes (Jeong *et al.* 2001) and we find that this is true irrespective of whether we are dealing with shared essentials or TRG essentials: not only do the essential proteins that are shared between yeast and worm have more interactions than other shared proteins, but essential TRPs also have more interactions than the nonessential TRPs (Figure 3A). Intriguingly, while both the shared essential proteins and TRPs have many interactions, they may achieve this in several different ways. First, while yeast and worm shared essential proteins tend to be highly structured, yeast- and worm-specific essential TRPs often contain large regions predicted to be disordered (Figure 3B and Figure S6). Crucially, in worms, essential TRPs have significantly more unstructured regions than nonessential TRPs, suggesting that this level of disorder is not specific to TRPs in general, but that these regions may contribute to their essential functions. It could be the case that this result is an artifact of excluding genes with well-annotated domains in the process of defining TRGs. However, repeating the analysis and including genes with well-annotated domains we find that it is still the case that yeast and worm essential TRPs have more disordered unstructured regions than shared essentials (yeast *P* < 10^−8^, worm *P* < 0.05), and that worm essential TRPs have more unstructured regions than nonessential TRPs (*P* < 10^−11^). Disordered regions are known to mediate many PPIs; some become highly ordered following protein–protein binding, others remain unstructured and act as a more flexible protein–protein interface than those between highly structured proteins. They may also act as a rapidly evolving source of PPI motifs (Goldman *et al.* 2014; Davey *et al.* 2015). In addition to this large increase in the degree of disorder in essential TRPs, we also find that both yeast and worm essential TRPs have more coiled-coil domains than nonessential TRPs (*P* < 0.01 in all cases, Figure 3C; χ^2^ test). Coiled coils also mediate PPIs and, like disordered regions, are easy to evolve *de novo* and have evolved many independent times (Rackham *et al.* 2010).

Taken together, the picture that emerges is that essential TRPs make a larger number of PPIs than nonessential TRPs, and that essential TRPs are enriched for both disordered regions and coiled coils. These are perhaps the easiest protein regions to evolve *de novo*, and the fastest evolving protein-coding regions in eukaryotic genomes.

### Many essential TRGs play roles in chromosome segregation

Our analyses above show that TRGs that have experimentally observed essential functions are distinct from nonessential TRGs in many ways, including differences in expression, in the interactions made by the proteins they encode, and in the domains that they contain. These analyses do not tell us anything concrete about the specific functions of these essential TRGs, however. “Guilt by association” is a powerful way to infer biological functions of uncharacterized genes (Lee *et al.* 2010) and thus we examined how essential TRGs interact with other genes using available large-scale functional data sets.

**Figure 4:**
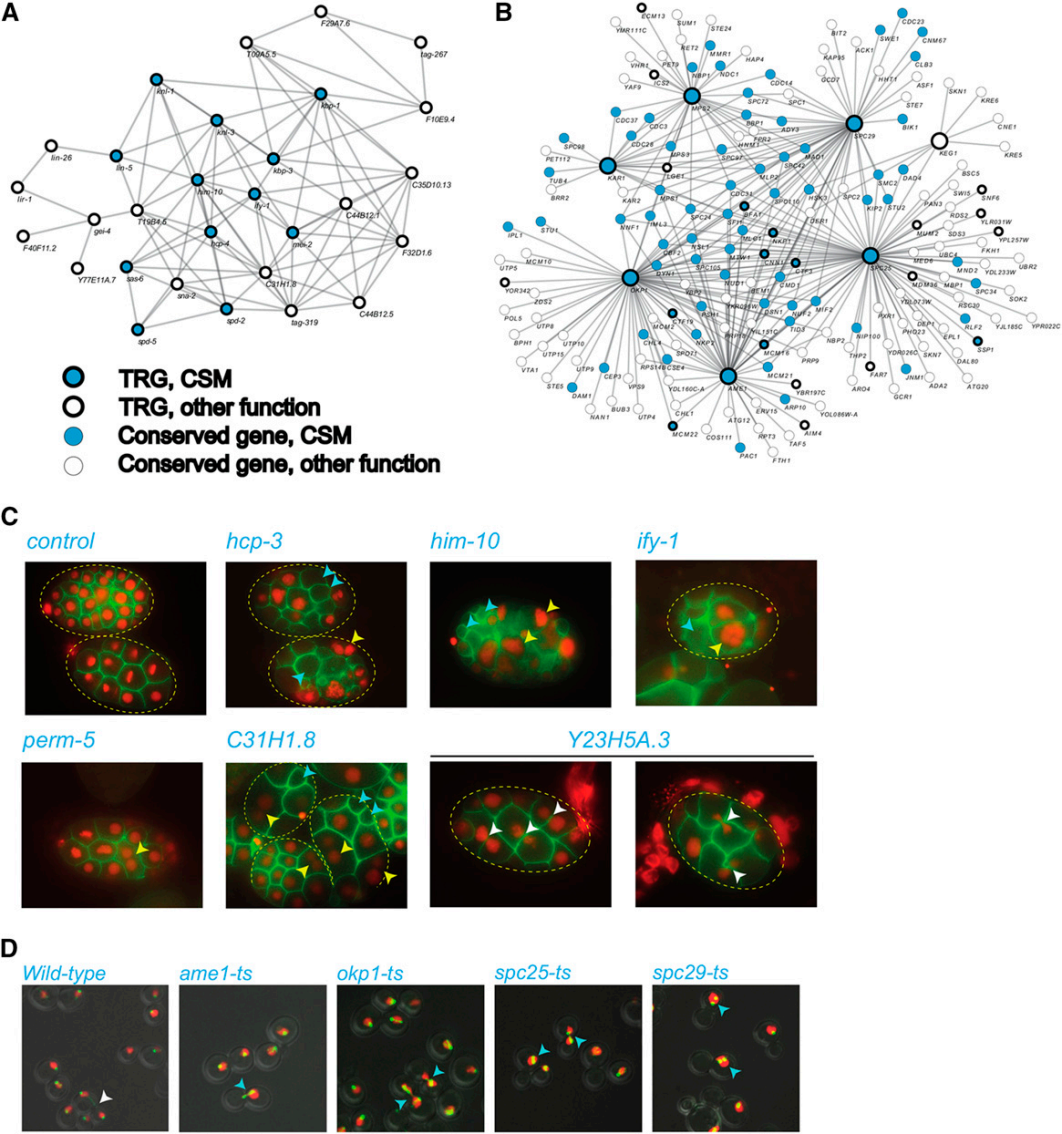
Essential TRGs have a biological role in chromosome segregation. (A) Wormnet connections for essential TRGs in *C. elegans*. Nodes which are TRGs are shown with a thick border; other genes have a thin border. Genes annotated with chromosome segregation GO terms which we found enriched in this set are colored in light blue, while other nodes are white. The Wormnet network shows the largest connected component of essential TRGs. (B) Yeastnet connections for essential TRGs in *S. cerevisiae*. The meaning of nodes and connections is the same as in (A). The Yeastnet network shows the largest connected component of essential TRGs as well as the top 100 connected genes. Essential TRGs are shown as enlarged nodes. (C) Targeting essential TRGs by RNAi results in chromosome-segregation defects in early embryos. Bacteria expressing dsRNA were fed to L4 animals that transgenically express fluorescent proteins marking chromatin (red) and the plasma membrane (green) for 72 hr, during which they developed into adults and laid embryos. Embryos were visualized by fluorescence microscopy. Nontargeting dsRNAs (control) had no effect on chromosome segregation, whereas targeting essential TRGs had penetrant chromosome-segregation defects leading to anucleate cells (blue arrowheads), multinucleate cells (yellow arrowheads), or chromatin bridges between dividing cells (white arrowheads). (D) Representative micrographs highlighting yeast cells with wild-type (top left panel) and aberrant spindle and spindle pole body morphologies (remaining panels). Dad2-GFP and Hta2-mCherry fluorescent signals are overlaid on DIC images, and cells of interest are indicated with white arrows (wild-type image) and blue arrows (mutants).

In yeast, PPI data are rich and PPIs can give a very direct view of how any protein may be acting. We clustered the yeast PPI network using an MCL (Enright *et al.* 2002), and found a single cluster which is significantly enriched for TRGs. Genes in this cluster are enriched for GO terms relating to chromosome structure and segregation (*e.g.*, “chromosome segregation,” “kinetochore,” “attachment of spindle microtubules to kinetochore”) (Figure S7 and Table S2), suggesting that TRGs in this cluster may have similar roles. In *C. elegans*, PPI data are much sparser than in yeast and thus we used a more diverse set of functional linkages between genes contained in Wormnet (Lee *et al.* 2008, 2010). Wormnet is a high-coverage network of functional linkages between genes that comprise coexpression, physical interactions, and genetic interactions among others; an analogous network (Yeastnet) exists for yeast (Kim *et al.* 2014). We find that in Wormnet, many essential worm TRGs interact and have functional connections to genes that have well-characterized roles in chromosome segregation, including *hcp-4*, *sas-6*, *kbp-1*, *spd-2*, and *spd-5* (Oegema and Hyman 2006), and indeed such chromosome segregation genes are enriched in this cluster (*P* < 10^−11^, hypergeometric test). We find similar results for essential TRGs in yeast using Yeastnet, and the local networks enriched for chromosome-segregation machinery in worm and yeast are shown in Figure 4, A and B. This finding that the essential TRGs are functionally linked to known components of the chromosome-segregation machinery concords well with published functional annotations: 10/23 yeast essential TRGs and 14/26 functionally annotated worm essential TRGs have annotated roles in chromosome maintenance and segregation (*P* < 0.0001 for both, hypergeometric test). Together, existing functional annotations, PPIs, and integrated functional networks all suggest that in both worms and in yeast, TRGs are enriched for essential functions in chromosome segregation.

To confirm these “guilt by association” functional predictions, we experimentally examined the loss-of-function phenotypes of a subset of 13 yeast and 12 worm essential TRGs. In *C. elegans*, we used RNAi to generate loss-of-function phenotypes, whereas in yeast we used temperature-sensitive alleles of our candidate genes. These results, shown in Figure 4, C and D, largely confirm the predictions: many of the yeast and worm essential TRGs that we examined have loss-of-function phenotypes that resemble those of well-characterized components of the chromosome-segregation machinery. For example, when we used RNAi to target either *hcp-3*, the well-characterized *C. elegans* CENP-A ortholog, or *him-10*, the highly diverged Nuf2 kinetochore ortholog, it resulted in embryos that have both multinucleate and anucleate cells (Figure 4C); we see a similar chromosome segregation-defective phenotype for the TRGs *perm-5*, *ify-1*, and *C31H1.8*. In the case of the TRG *Y23H5A.5*, the RNAi phenotype shows aberrant chromosome segregation leading to chromatin “bridges” between dividing cells (Figure 4C). In yeast, strains carrying temperature-sensitive alleles of TRGs including the spindle pole body component *SPC29* and the essential kinetochore components *AME1* and *OKP1*, for example, also have extensive chromosome-segregation defects when grown at the nonpermissive temperature (Figure 4D). In yeast, these genes were previously known to have a role in chromosome segregation, but in worm we have characterized new phenotypes for *perm-5*, *C31H1.8*, and *Y23H5A.5*. These experiments confirm that many yeast and worm essential TRGs play critical roles in chromosome segregation, a strong enrichment compared with shared essentials which play roles in a much more diverse set of cellular processes.

It is possible that the results we present in this article are dependent on the point on the phylogenetic tree at which we chose to define TRGs. To ensure our results are robust to such choices, we have repeated our analyses defining TRGs at the more restricted level of *Caenorhabditis* rather than at the level of Chromadorea. We still find that essential TRGs have expression profiles similar to that of shared essential genes (as in Figure 2B, data not shown, *P* < 10^−15^). Furthermore, the expression is higher in genes surrounding essential TRGs (as in Figure 2C, data not shown, *P* < 0.001), and essential TRGs are still enriched in regions of active chromatin (as in Figure 2D, data not shown *P* < 10^−8^). With this narrower definition of TRGs, it is still the case that essential TRPs have more PPIs (as in Figure 3A, *P* < 0.01), they have more disordered regions (as in Figure 3B, *P* < 10^−6^), and they have more coiled-coil regions (as in Figure 3C, *P* < 10^−4^). Therefore, our results are robust to the way we have defined TRGs, providing confidence to our results.

## Discussion

Many of the genes encoded in any genome have no identifiable orthologs outside very closely related species. These TRGs are largely unstudied and are poorly functionally characterized. In this study, we examined the functions of TRGs in two eukaryotes, the budding yeast *S. cerevisiae* and the nematode worm *C. elegans*. We find that in both species, essential TRGs have many similar molecular properties to those of highly conserved essential genes: they are expressed similarly, and their encoded proteins have a high number of PPIs. However, unlike shared essential genes, they encode proteins with a greater degree of disorder, suggesting that they make these interactions in different ways. Interestingly, we find that many TRGs play essential roles in chromosome segregation, a core cellular process, and suggest that this is driven both by selection for divergence of existing components and through the recruitment of novel components to the chromosome-segregation machinery. We thus propose that while TRGs can underlie the evolution of lineage-specific behaviors and structures, they may also play lineage-specific central roles in shared processes such as chromosome segregation. TRGs can thus drive both the things you can do, and the way that you do them.

Our finding that many of the essential lineage-specific TRGs in yeast and worm play key roles in the chromosome-segregation machinery is intriguing. Chromosome segregation is one of the most basic cellular functions; all cells must be able to carry this out correctly and efficiently. Why should so many TRGs play key roles in this core process?

**Figure 5:**
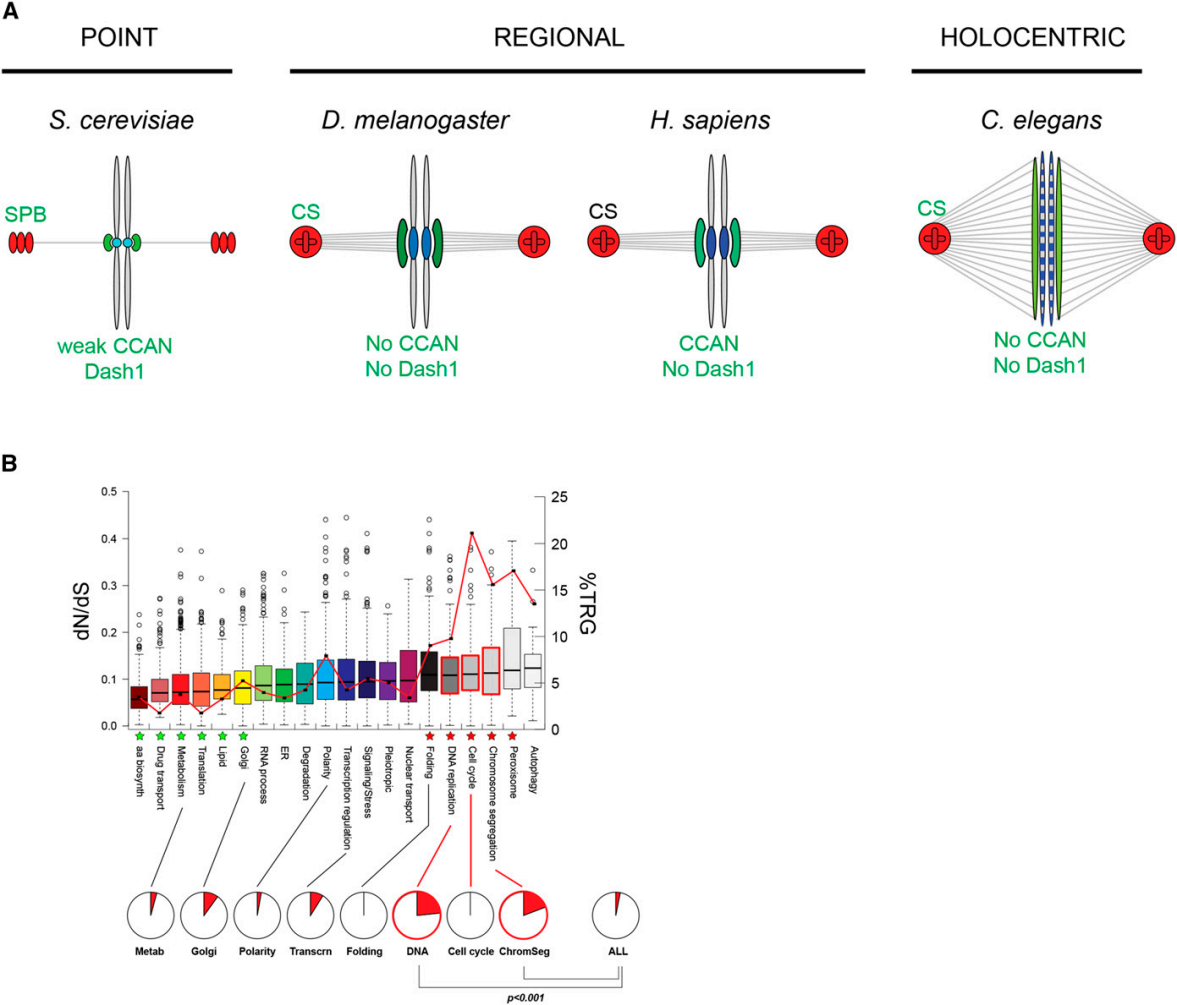
Essential TRGs often have a role in chromosome separation. (A) A schematic showing differences in chromosome-segregation machinery in different opisthokonts. Spindles are shown in gray, microtubule-organizing centers are shown in shades of red, kinetochores in shades of green, and centromeric regions in shades of blue. Key kinetochore complexes acting in chromosome segregation are listed for each species. (B) Rate of evolution of different functional classes of *S. cerevisiae* genes. The box plot shows the distribution of dN/dS values for yeast genes in each of 19 functional classes. Classes with significantly lower dN/dS are marked with green ☆; classes with higher dN/dS are marked with red ☆. The percentage of genes in each class that are TRGs is shown by the red line graph; functional classes with significantly higher numbers of TRGs are highlighted in red text. Pie charts show the proportion of TRGs that have essential functions in all classes that have at least 20 TRGs. Functional classes are from Costanzo *et al.* (2010), and dN/dS data are from Scannell *et al.* (2011).

At its core, the chromosome-segregation machinery must nucleate microtubules from a microtubule-organizing center to form a spindle; spindles are then attached to chromosomes at centromeric sites via the kinetochore which acts as the adaptor between the spindle and the DNA. Surprisingly, centromeres, kinetochores, and microtubule-organizing centers are highly variable and different animal species have different solutions to the problem of chromosome segregation (see Figure 5A) (Azimzadeh 2014; Duro and Marston 2015). The centromere is one of the most rapidly evolving DNA sequences, and its structure and chromosomal location vary widely between species (Malik and Henikoff 2009). Some species, like *S. cerevisiae*, have a short (∼125 bp) point centromere; others like humans and *Schizosaccharomyces pombe* have a regional centromere comprising several hundred kilobases; and finally, some species like *C. elegans* are holocentric, with spindle-attachment points along the entire length of the chromosome. Centromeric DNA is usually assembled into nucleosomes that contain the CENP-A histone H3 variant and, unlike other histones, this is rapidly evolving under positive selection in *Arabidopsis* (Talbert *et al.* 2002), *Drosophila* (Malik and Henikoff 2001), and primates (Schueler *et al.* 2010). There are also other genes which are also rapidly evolving roles at the centromere such as the *Umbrea* gene in *Drosophila* (Ross *et al.* 2013). Kinetochores assemble at centromeric regions and these multiprotein complexes also differ greatly between species, containing many TRGs. For example, *S. cerevisiae* kinetochores require the fungal-specific Cbf3 and DASH complexes (Meraldi *et al.* 2006); while, in humans, kinetochores require the vertebrate-specific SKA complex (Welburn *et al.* 2009); and the CCAN complex that is essential in humans is absent from fly and nematode genomes (Westhorpe and Straight 2013). Finally, the microtubule-organizing center also varies greatly between animal cells: while animals use centriole-containing centrosomes, yeasts use a completely different structure, the spindle pole body; there is no homology between most of the components of these structures.

The centromeres, kinetochores, and microtubule-organizing centers all differ greatly between metazoan species—is this rapid evolution typical in all cellular machineries? We examined rates of evolution of a broad set of functional classes of yeast genes and find that genes that function in chromosome segregation are of the most rapidly evolving classes of gene (Figure 5B). We also find that the functional classes that show the most rapid divergence of shared genes are also the functional classes that include the highest proportion of TRGs. For example, the amino acid biosynthesis pathway has the lowest rate of divergence (median dN/dS = 0.057; *P* < 0.0001, *t*-test) and also one of the lowest proportions of TRGs (3.5%; 7/200); the chromosome-segregation machinery, on the other hand, has one of the highest rates of divergence (median dN/dS = 0.113; *P* < 0.005, *t*-test) and the highest proportions of TRGs (15.6%; 36/231). We note that the picture that emerges from yeast and nematodes of the chromosome-segregation machinery as evolving rapidly and containing many TRGs appears to hold across the eukaryotic tree. For example, the kinetochores of *Trypanosoma brucei*, a member of class Kinetoplastida, are comprised almost entirely of TRGs (Akiyoshi and Gull 2014). The chromosome-segregation machinery thus appears to be particularly enriched for TRGs, its components are diverging rapidly, and the mechanisms of chromosome segregation are hugely diverse across eukaryotes (Drechsler and McAinsh 2012).

Why might it be that we find so many essential TRGs in the chromosome-segregation machinery of different lineages? TRGs arise either by the *de novo* evolution of genes, or through the lineage-specific divergence of ancestral ortholog genes to the point of unrecognizable homology. It has previously been observed that some chromosome-segregation machinery components diverge rapidly at the sequence level through positive selection; in this model, centromeres are selfish drivers and are thus subject to “arms races” much like host parasite conflicts (Henikoff and Malik 2002). We think this is unlikely to be the explanation for the high number of essential TRGs that we observe to participate in the chromosome-segregation machinery in yeast and nematodes. There is no evidence of positive selection in the TRGs we examined: the dN/dS is substantially less than one in all cases, indicating that all these genes are under purifying selection. Moreover, the mechanics of chromosome-segregation machinery in nematodes and yeast do not support a selfish driver model; in nematodes, chromosomes are holocentric, whereas in yeast meiosis yields four viable haploid spores. In neither case can meiotic drive result in selection for chromosome segregation-machinery divergence.

We suggest that the large numbers of apparently lineage-specific genes in the chromosome-segregation machinery are because a very wide range of sequences must be able to carry out similar essential functions in chromosome segregation. TRGs in the chromosome-segregation machinery are either under weak selection, allowing them to drift rapidly until homology is unrecognizable, even over relatively short evolutionary distances; or some of the functions of chromosome segregation-machinery components may simply be very easy to evolve and thus TRGs that arise *de novo* can readily evolve roles in this process. For example, eukaryotic microtubule-organizing centers are very diverse and range from evolutionarily unrelated protein complexes (*e.g.*, the spindle pole body in yeast and centrosomes in animals), to membranes (*e.g.*, the nuclear envelope in plants), to condensed DNA structures in kinetoplastids. This diversity shows that many different structures can act as effective microtubule-organizing centers and that this is a readily evolvable function. Similarly, many kinetochore components consist largely of coiled-coil regions; these evolve *de novo* very readily (Rackham *et al.* 2010) and novel coiled-coil domains could acquire roles in this complex. We note that convergent evolution occurs frequently in proteins that have no enzymatic activity but act as multimerized complexes, such as antifreeze proteins (Chen *et al.* 1997a) and the crystallins that make up eye lenses (Vopalensky and Kozmik 2009). The microtubule-organizing centers and kinetochore might be similar: they are organized in large multimeric complexes, and much of their molecular function rests on protein interactions mediated by simple motifs like coiled coil and disordered regions. We therefore suggest that the great enrichment of essential TRGs in the chromosome-segregation machinery is driven by two factors: first, by very relaxed constraints on the sequences that can function in the chromosome-segregation machinery; and, second, by the ease with which novel components can evolve functions in the chromosome-segregation machinery through the *de novo* evolution of low complexity protein sequences such as coiled coils and disordered regions.

## Supplementary Material

Supplemental material is available online at www.g3journal.org/lookup/suppl/doi:10.1534/g3.117.300193/-/DC1.
