# Supplementary material for: Taxonomically Restricted Genes with Essential Functions Frequently Play Roles in Chromosome Segregation in Caenorhabditis elegans and Saccharomyces cerevisiae
Source: G3 (Bethesda). 2017 Aug 24;7(10):3337–47. doi: 10.1534/g3.117.300193 (PMC5633384; doi:10.1534/g3.117.300193)
Supplement: Supplementary file 10 [file 3337FileS1.docx]

**Supporting Information Legends**

**Fig. S1. The fraction of essential TRGs which are misannotated with different BLAST cutoffs.** On the x-axis we change the Evalue cutoff, here shown as the –log_10_ of the Evalue cutoff, while curves of different colors show how changing the minimum percent length of the hit affects our results.

**Fig. S2. Phylogenetic distribution of BLAST scores for 21 yeast essential TRGs.** Each plot shows the –log_10_ BLASTP Evalue for the highest BLAST match of each *S. cerevisiae* gene in a range of eukaryote genomes including those of the closely related yeasts *Vanderwaltozyma polyspora (VP), Naumovia castelli (NC), Zygotosaccharomyces rouxii (ZR), Tetrapispora phaffii (TP), Debaryomyces hansenii (DH), Candida albicans (CA),* more distantly related yeasts *Clavispora lusitaniae (CL)*, *Yarrowia lypolytica (YL), Apergillus fumigatus (AF)* and *Schizosaccharomyces pombe* as well as the fungus *Coprinopsis cinerea,* the slime mould *Dictostelium discoidum* and the animals *C. elegans, D. melanogaster* and *H. sapiens.* Genomes are ordered bottom to top from closest to furthest from *S. cerevisiae* and the dotted line marks the cut-off used to define TRGs. Two yeast essential TRGs are not shown since there was no significant homology in other genomes. The yeast phylogeny is taken from (Suh, Blackwell, Kurtzman, & Lachance, 2006). The last common ancestor of species of interest are marked on the phylogeny as colored circles. Numbers above the branch or slash indicate 50% bootstrap support while the numbers below the branch or slash indicate the probability of nodes in a Bayesian analysis. The species in bold indicate the type species of each genus.

**Fig. S3. *S.cerevisiae* essential genes have different molecular properties compared to genes without reported essential phenotypes.** We show the distribution of dN/dS values, dN values and expression levels for either *S.cerevisiae* essential genes (Giaever et al., 2002) or genes without clear essential phenotypes. dN/dS data all from (Scannell et al., 2011) and expression data from (Lipson et al., 2009).

**Fig. S4. Expression correlation of TRGs to shared pathways is predictive of having an essential phenotype in *S. cerevisiae*.** Shown here is the distribution of the average expression correlation to genes in KEGG pathways sce03020 (RNA polymerase), sce03040 (Spliceosome) and sce03050 (Proteasome) for essential and non-essential TRGs as a density plot. Correlations have been Z transformed to improve comparability between datasets. Stars correspond to significance assessed using a Wilcox rank sum test.

**Fig. S5. Metrics for the logistic regression classifier used to predict which TRGs have essential phenotypes. (A) Cross validation prediction ROC curve of TRG essentiality.** We trained a classifier to discriminate between essential and non-essential TRGs based on their expression across a diverse set of developmental and environmental conditions. The area under the ROC curve calculated in cross-validation is 0.87. **(B) The proportion of essential genes in any functional module correlates with the predictive power of that module.** This graph is showing individual feature prediction ROC value against the percentage of genes in that feature which are essential. The correlation is 0.42.

**Fig. S6. Predicted protein disorder in TRPs and shared proteins in *C. elegans* and *S. cerevisiae*.** This plot is similar to Fig. 3B, but with X-ray disorder instead of NMR mobility disorder from Espritz. Shared-E is essential shared proteins; Shared-NE is non-essential shared proteins; TRP-E is essential TRPs; TRP-NE is non-essential TRPs.

**Fig. S7. A *S. cerevisiae* PPI cluster enriched for TRGs.** The yeast protein-protein interaction data were clustered using markov clustering with an inflation factor of 1.5. The single cluster found is shown here with kinetochore annotated genes in blue and TRGs with a thick border.

**Table S1. TRGs identified in *C. elegans* and *S. cerevisiae*.** A detailed description of how we identified TRGs in these genomes and how genes were defined as essential is found in the methods section.

**Table S2. GO Enrichment for the *S. cerevisiae* PPI network cluster identified using MCL.** Here we show the GO ID, GO name, bonferonni adjusted p-value, the enrichment factor which is the ratio of proportion of genes in the cluster to the ratio of genes in the background and the percentage of genes in the cluster with that term.

**References**

Giaever, G., Chu, A. M., Ni, L., Connelly, C., Riles, L., Véronneau, S., … Johnston, M. (2002). Functional profiling of the Saccharomyces cerevisiae genome. *Nature*, *418*(6896), 387–391. https://doi.org/10.1038/nature00935

Lipson, D., Raz, T., Kieu, A., Jones, D. R., Giladi, E., Thayer, E., … Causey, M. (2009). Quantification of the yeast transcriptome by single-molecule sequencing. *Nature Biotechnology*, *27*(7), 652–658. https://doi.org/10.1038/nbt.1551

Scannell, D. R., Zill, O. A., Rokas, A., Payen, C., Dunham, M. J., Eisen, M. B., … Hittinger, C. T. (2011). The Awesome Power of Yeast Evolutionary Genetics: New Genome Sequences and Strain Resources for the Saccharomyces sensu stricto Genus. *G3*, *1*(1), 11–25. https://doi.org/10.1534/g3.111.000273

Suh, S.-O., Blackwell, M., Kurtzman, C. P., & Lachance, M.-A. (2006). Phylogenetics of Saccharomycetales, the ascomycete yeasts. *Mycologia*, *98*(6), 1006–17. Retrieved from http://www.ncbi.nlm.nih.gov/pubmed/17486976
